# Metastatic Exophytic Gastric Adenocarcinoma Diagnosed Using Endoscopic Ultrasound-Guided Fine-Needle Biopsy: A Case Report

**DOI:** 10.7759/cureus.105616

**Published:** 2026-03-21

**Authors:** Yoshifumi Watanabe, Yasumasa Sumitomo

**Affiliations:** 1 Department of Gastroenterology, Hoshigaoka Medical Center, Hirakata, JPN

**Keywords:** adenocarcinoma, endoscopic ultrasonography, fine-needle biopsy, gastric cancer, gastrointestinal stromal tumor, subepithelial lesion

## Abstract

A 75-year-old female patient presented to our department with abdominal distension for the past month. Physical examination revealed abdominal distension without tenderness. Carcinoembryonic antigen, carbohydrate antigen 19-9, and cancer antigen 125 levels were 4.2 ng/mL, 125.8 U/mL, and 8,423.9 U/mL, respectively. Contrast-enhanced computed tomography displayed a 110-mm-sized exophytic gastric tumor. The tumor was accompanied by ascites but without lymph node enlargement. Esophagogastroduodenoscopy revealed extrinsic compression in the posterior wall of the gastric fundus. The surface was completely covered with normal mucosa, and endoscopic forceps biopsy exhibited no abnormal findings. The cytodiagnosis of ascites obtained by abdominal paracentesis revealed adenocarcinoma. Endoscopic ultrasonography revealed that the exophytic gastric tumor was uniformly hypoechoic, and a fine-needle biopsy was performed. The histopathological finding revealed adenocarcinoma. Immunohistochemical analysis showed that these cells expressed cytokeratin 7 but not cytokeratin 20, CD117, CD34, chromogranin A, and synaptophysin, confirming the diagnosis of exophytic gastric adenocarcinoma with peritoneal dissemination. The tumor was mismatch repair proficient and did not express claudin-18.2 and human epidermal growth factor receptor 2. Programmed death-ligand 1 combined positive score was <10. Based on the results of genetic expression, chemotherapy comprised of tegafur-gimeracil-oteracil potassium, oxaliplatin and nivolumab was considered; however, the patient rapidly deteriorated and died of progression of underlying cancer 39 days following the initial visit to our hospital.

## Introduction

Gastric cancer represents the fifth most common malignant tumor and the fifth leading cause of cancer-related deaths worldwide [1]. Adenocarcinoma accounts for approximately 90% of all gastric cancer [2]. Gastric cancer originates from mucosal epithelial cells and generally grows, forming a polypoid, ulcerated, or diffusely infiltrative lesion [2]. The diagnosis can be made by endoscopic forceps biopsy from the surface of the lesions [2]. Gastric cancer, which spreads further below the submucosa compared to the mucosa, rarely presents like the form of subepithelial lesion [3]. Gastric cancer with features of subepithelial lesion (GCSEL) accounts for 0.2%-0.62% of all gastric cancer [3]. The main issue associated with GCSEL is establishing the definitive diagnosis as the endoscopic biopsy diagnosis rate of GCSEL is 47.4% [4]. In GCSEL without erosion or ulcer, pathological diagnosis using conventional endoscopic forceps biopsy is difficult, as the tumor is situated within the gastric wall and is covered with normal mucosa; however, conventional endoscopic forceps biopsy from mucosal erosion or ulceration typically facilitates diagnosis [4]. Boring biopsy and endoscopic ultrasound-guided tissue acquisition (EUS-TA) are performed in GCSEL without erosion or ulcer [4]. Notably, endoscopic ultrasound-guided fine-needle biopsy (EUS-FNB) is expected to enable accurate diagnosis and analysis of molecular mutation status. Among GCSEL, exophytic gastric cancer, which grows outward from the gastric wall into the peritoneal cavity, is extremely rare; to the best of our knowledge, no cases wherein EUS-FNB was used for diagnosing exophytic gastric cancer have been reported. We here present a case of metastatic exophytic gastric adenocarcinoma diagnosed using EUS-FNB and review the relevant literature.

## Case presentation

A 75-year-old Japanese female patient presented to our department with abdominal distension for the past month. The patient had a history of chronic heart failure and insomnia. Physical examination revealed abdominal distension without tenderness. Laboratory examination demonstrated carcinoembryonic antigen, carbohydrate antigen 19-9, and cancer antigen 125 levels of 4.2 ng/mL, 125.8 U/mL, and 8,423.9 U/mL, respectively (Table 1).

**Table 1:** Laboratory findings on admission ALT: Alanine aminotransferase; AST: Aspartate aminotransferase; ALP: Alkaline phosphatase; LDH: Lactate dehydrogenase; CEA: Carcinoembryonic antigen; CA19-9: Carbohydrate antigen 19-9; CA125: Cancer antigen 125

Parameter	Measured value	Reference range
White blood cell (×10⁹/L)	5.8	3.3-8.9
Hemoglobin (g/L)	124	114-146
Platelet (×10⁹/L)	264	140-359
Bilirubin (µmol/L)	13.7	3.4-18.8
ALT (U/L)	9	5-35
AST (U/L)	24	5-35
ALP (U/L)	58	38-113
Albumin (g/L)	30	39-49
LDH (U/L)	305	124-222
Creatinine (µmol/L)	47.7	44.2-70.7
Sodium (mmol/L)	127	135-147
Potassium (mmol/L)	4.8	3.5-5.1
C-reactive protein (mg/L)	55.3	0-3
CEA (ng/mL)	4.2	0-5
CA19-9 (U/mL)	125.8	0-37
CA125 (U/mL)	8423.9	0-35

**Figure 1:**
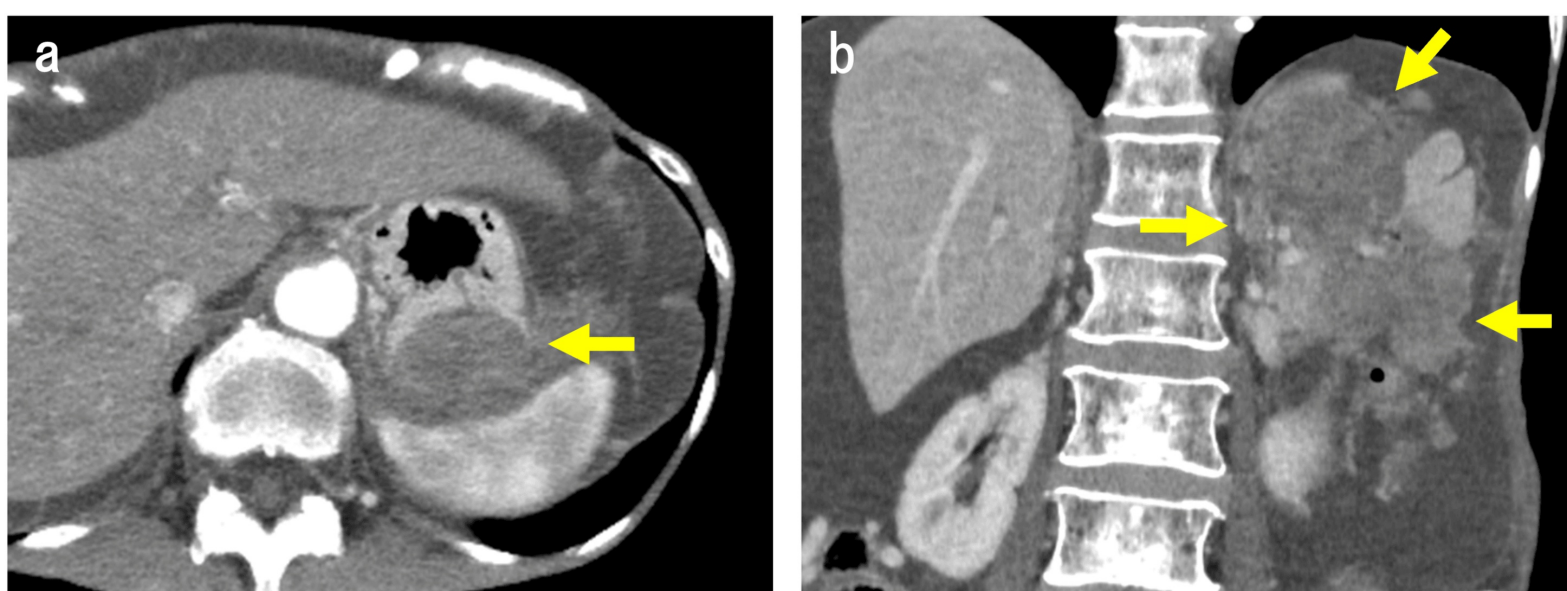
Contrast-enhanced computed tomography images (a) A 110-mm-sized exophytic gastric tumor and ascites. The gastric wall adjacent to the exophytic gastric tumor is discontinuous; however, the continuity of the gastric intraluminal surface is maintained (arrow). (b) Coronal image of exophytic gastric tumor (arrows).

Contrast-enhanced computed tomography (CT) revealed a 110-mm-sized exophytic gastric tumor (Figure 1a, 1b).

**Figure 2:**
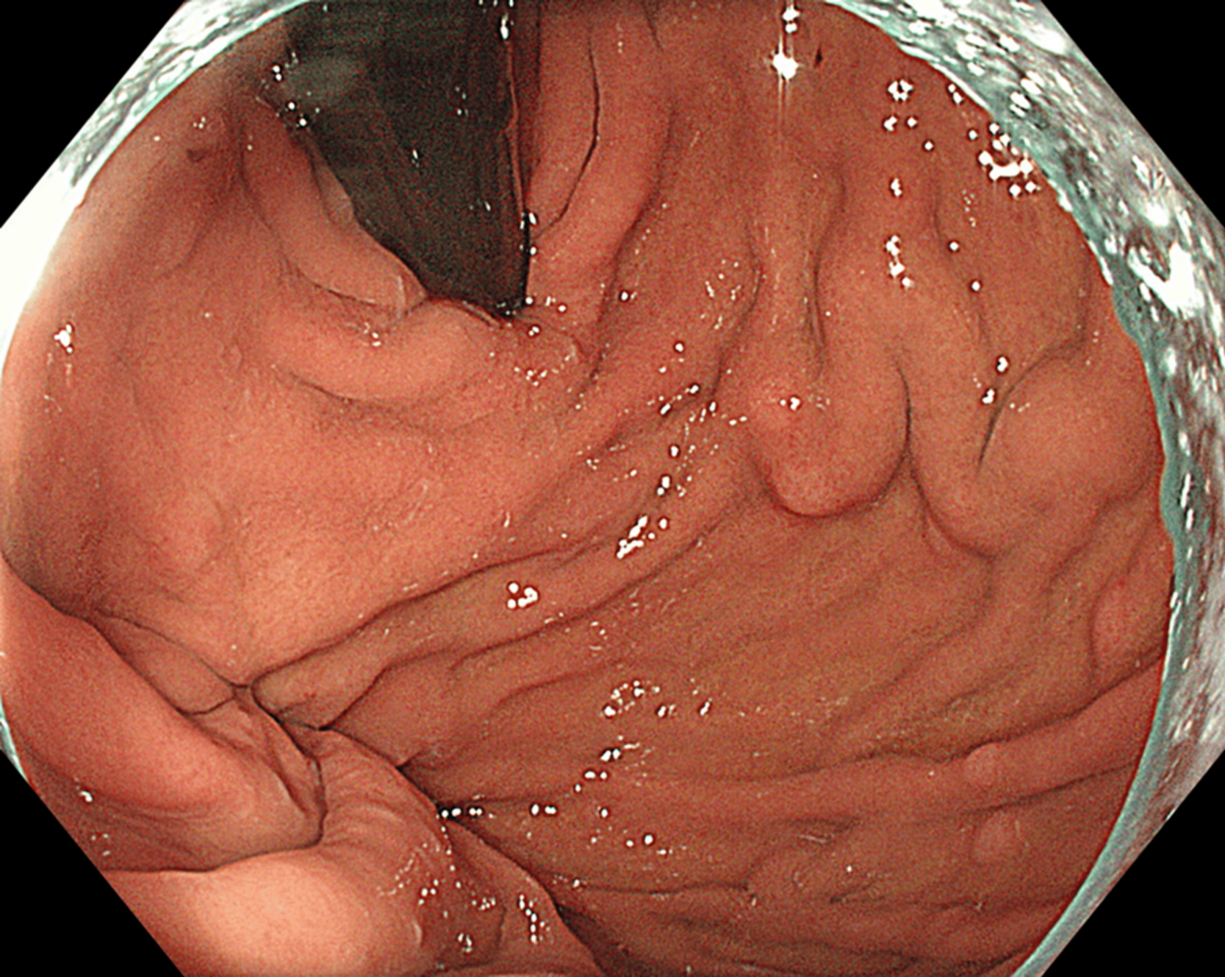
Esophagogastroduodenoscopy images The posterior wall of the gastric fundus is compressed extrinsically without erosion or ulcer.

The gastric wall adjacent to the exophytic gastric tumor was discontinuous; however, the continuity of the gastric intraluminal surface was maintained (Figure 1a). The tumor was accompanied by ascites but without lymph node enlargement. Esophagogastroduodenoscopy revealed extrinsic compression in the posterior wall of the gastric fundus (Figure 2).

**Figure 3:**
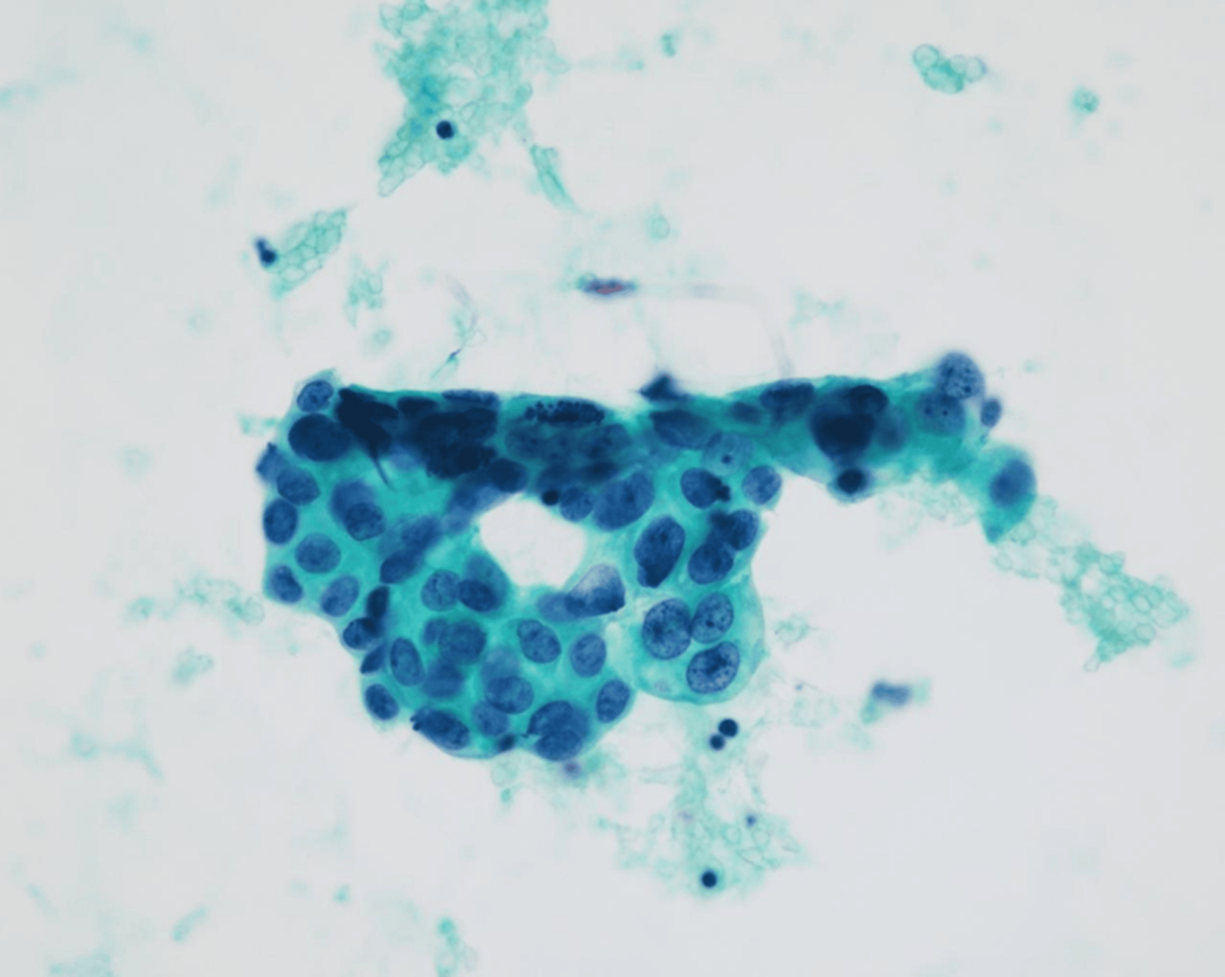
Cytological image obtained using abdominal paracentesis Cytodiagnosis of ascites reveals adenocarcinoma.

The surface was completely covered with normal mucosa without erosion or ulcer, and endoscopic forceps biopsy showed no malignant findings. Moreover, colonoscopy results were not significant. Abdominal paracentesis was performed, and the cytodiagnosis of ascites revealed adenocarcinoma (Figure 3).

**Figure 4:**
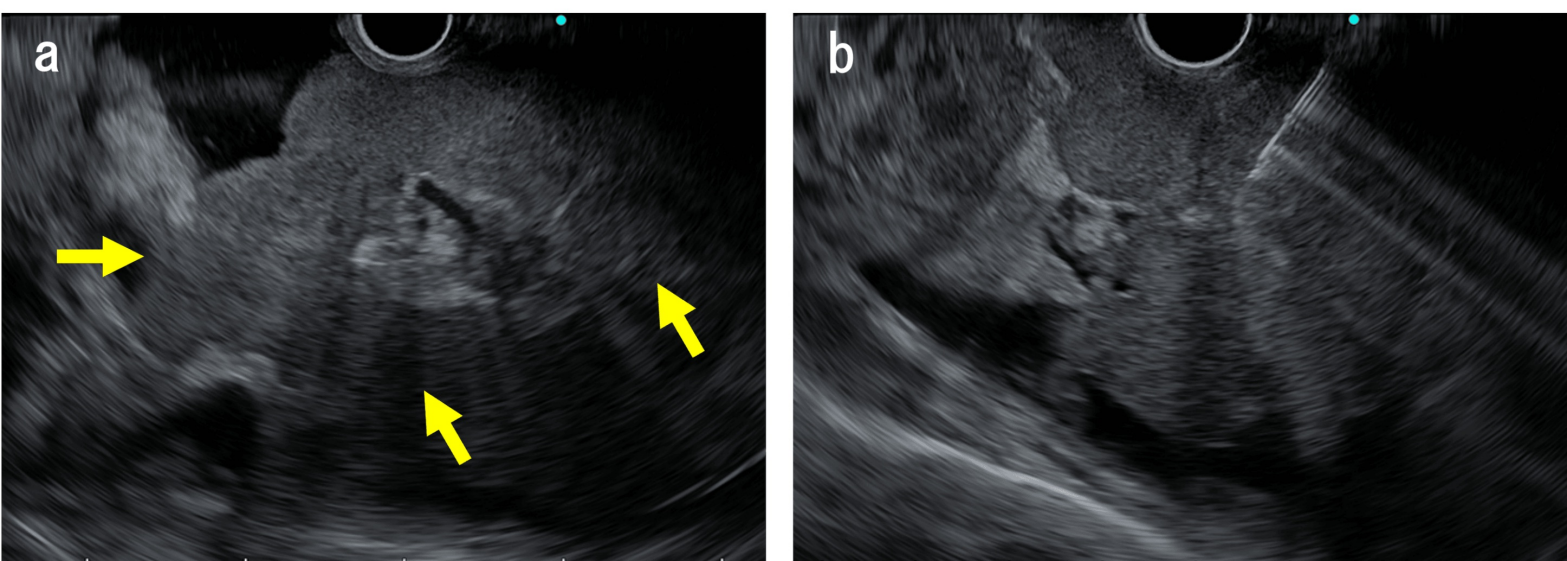
Endoscopic ultrasonography images (a) The exophytic gastric tumor showing a uniformly hypoechoic mass (arrows). (b) Endoscopic ultrasound-guided fine-needle biopsy is performed for definitive diagnosis.

Endoscopic ultrasonography revealed that the tumor presented as a uniformly hypoechoic mass (Figure 4a).

**Figure 5:**
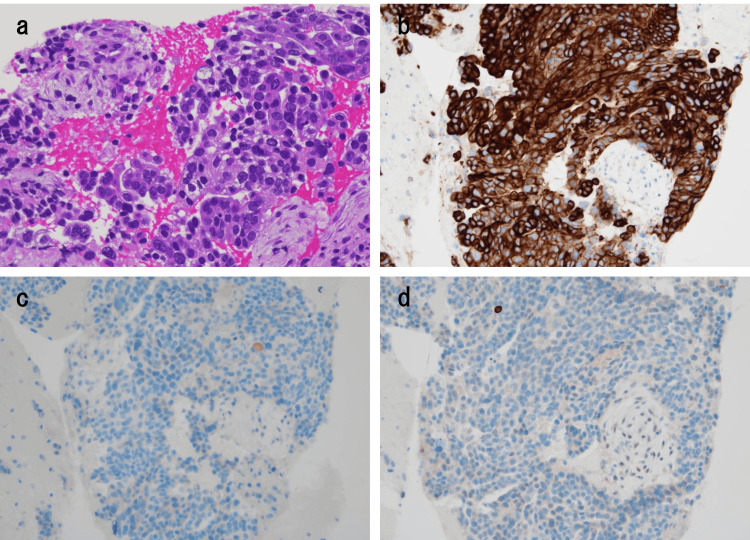
Histopathological examination of exophytic gastric tumor specimens obtained using endoscopic ultrasound-guided fine-needle biopsy (a) The tumor is identified as adenocarcinoma in microscopic examination (hematoxylin and eosin staining). On immunohistochemical analysis, tumor cells express (b) cytokeratin 7 and not (c) cytokeratin 20 and (d) CD117. The results were consistent with the typical findings that most gastric adenocarcinoma is positive for CK7, whereas GIST is positive for CD117 immunohistochemically.

EUS-FNB using a 22-gauge Trident needle (Micro-Tech Endoscopy, High Wycombe, UK) was performed for the definitive diagnosis of exophytic gastric tumor (Figure 4b). The specimen was identified as adenocarcinoma (Figure 5a).

Immunohistochemical analysis indicated that the cells expressed cytokeratin 7 but not cytokeratin 20, CD117, CD34, chromogranin A, and synaptophysin (Figure 5b-5d). These findings were consistent with the diagnosis of exophytic gastric adenocarcinoma with peritoneal dissemination. Furthermore, the molecular mutation status of the gastric adenocarcinoma was investigated. The tumor was mismatch repair proficient but did not express claudin-18.2 and human epidermal growth factor receptor 2 (HER2). Programmed death-ligand 1 (PD-L1) combined positive score was <10. Based on the results of genetic expression, chemotherapy comprised of tegafur-gimeracil-oteracil potassium, oxaliplatin and nivolumab was considered; however, the patient rapidly deteriorated and died of progression of underlying cancer 39 days following the initial visit to our hospital.

## Discussion

Exophytic gastric cancer cells predominantly infiltrate in the serosal direction and form an extraluminal tumor. Owing to the limited number of reported cases, only a few characteristics have been described [5,6]. The prognosis is poor because the cancer is frequently asymptomatic and advanced at diagnosis; however, no characteristic histological findings in terms of differentiation are noted [5]. Exophytic gastric cancer has a tendency to arise in the antrum [5,6]. In our case, the tumor was detected in the upper stomach, making the diagnosis difficult. The differential diagnosis based on imaging tests is gastric subepithelial lesion, primarily gastrointestinal stromal tumor (GIST), which is the most common among gastric mesenchymal neoplasms [6,7]. Most GISTs originate from the muscularis propria and grow exophytically [7]. GISTs tend to develop in the upper stomach and have the potential to become malignant [6].

Differentiating exophytic gastric cancer from extraluminal GIST in the diagnosis is crucial because the treatment varies between them [7,8]. Antral location, thickening of the gastric wall adjacent to an exogastric mass, and lymph node enlargement are the characteristic CT findings of exophytic gastric cancer [6]. Gastric wall thickening indicates that cancer has invaded the adjacent gastric wall [6]. More than half of patients with advanced gastric cancer develop lymph node metastasis, whereas lymph node metastasis of GIST is rare with a 1.66% incidence; lymph node dissection can be omitted in surgery for GIST [7-10]. Additionally, gastric cancer frequently presents with ascites when it disseminates to the peritoneum, whereas ascites is infrequent in GIST [11,12]. Our case exhibited no lymph node enlargement; however, the presence of ascites led to suspicion that the exophytic gastric tumor was cancer. However, for a definitive diagnosis of gastric tumor, pathological diagnosis is essential owing to limitations in the diagnostic accuracy rate of CT.

Gastric cancer cells shed into the peritoneal space and develop into peritoneal metastasis. Synchronous gastric peritoneal metastases were noted in approximately 10% of cases, and peritoneal recurrence following curative resection for gastric cancer occurred in 13.46% of cases [2,11]. GIST also metastasizes to the peritoneum; however, ascites occurrence in GIST is rare compared with that in gastric cancer [12]. This difference is because GIST does not generally generate an inflammatory response [12]. Abdominal paracentesis is frequently performed in patients with ascites; however, differentiating between GIST and adenocarcinoma is occasionally challenging using ascites cytology as GIST may demonstrate cytologic features strikingly similar to those of adenocarcinoma in ascitic fluid [13]. Therefore, a pathological diagnosis of the primary gastric tumor is crucial despite adenocarcinoma is detected on ascites cytology.

EUS-TA is a well-established technique for sampling specimens, and a definitive diagnosis was obtained in 81% of cases wherein previous biopsies using other techniques had been inconclusive [14]. Recently, FNB needles, which have a specially designed tip for improved tissue sampling, have become widely used. EUS-FNB has two advantages. First, it significantly contributes to an accurate diagnosis. The diagnosability of subepithelial lesion using FNB needles is 82%-92%, which is better than that of endoscopic ultrasound-guided fine-needle aspiration (EUS-FNA) needles [15,16]. Second, FNB needles can obtain better quality and greater quantity of specimens [17]. EUS-FNB provides more than twice the volume of tissue and more nucleic acid than EUS-FNA [17]. It enables immunohistochemical and genome analysis that can optimize beneficial individualized chemotherapy. Elhanafi et al. reported that EUS-FNB was identified as an independent factor contributing to the success of genomic analysis in pancreatic cancer [18]. Based on genetic mutations, including PD-L1, HER2, and claudin-18.2, microsatellite instability and mismatch repair status, chemotherapy regimen for patients with metastatic gastric cancer is determined [8]. Therefore, EUS-FNB is crucial in metastatic exophytic gastric cancer for diagnosing and individualizing chemotherapeutic strategies. The uniqueness of our case could be attributed to exophytic gastric cancer and performing EUS-FNB for diagnosis and molecular mutation status analysis.

## Conclusions

Exophytic gastric cancer is rare and challenging to diagnose using conventional endoscopic forceps biopsy. EUS-FNB is crucial for definitive diagnosis as CT has limitations in differentiating exophytic gastric cancer from GIST. Recently, chemotherapy for metastatic gastric cancer has become individualized based on genetic mutations, and the quantity and quality of specimens for immunohistochemical and genome analyses are more critical than ever. The specimens obtained using EUS-FNB are suitable for the analysis. EUS-FNB may represent the most reliable approach for definitive diagnosis and a promising treatment strategy in exophytic gastric cancer. A large number of cases must be accumulated to establish optimal management.
